# Announcement of the Starling Medical College, Columbus, Ohio

**Published:** 1869-06

**Authors:** 


*Announcement of the Starling Medical College, Columbus, Ohio.*—The lectures in this college commence October 20th, and continue to the first of March. The institution is in a prosperous condition, as shown by catalogue of students. Several new men have been added to the Faculty, and as now organized all branches of medical science are to be thoroughly taught.

